# Dimensionality analysis of the German version of the Screen for Cognitive Impairment in Psychiatry (SCIP-G)

**DOI:** 10.1016/j.scog.2022.100259

**Published:** 2022-06-06

**Authors:** Gabriele Sachs, Gloria Bannick, Eva I.J. Maihofer, Martin Voracek, Scot E. Purdon, Andreas Erfurth

**Affiliations:** a Medical University of Vienna, Vienna, Austria; b 1^st^ Department of Psychiatry and Psychotherapeutic Medicine, Klinik Hietzing, Vienna, Austria; c Department of Cognition, Emotion, and Methods in Psychology, Faculty of Psychology, University of Vienna, Vienna, Austria; d Alberta Hospital Edmonton Neuropsychology, Department of Psychiatry, University of Alberta, Edmonton, Alberta, Canada

**Keywords:** Schizophrenia, Screen for Cognitive Impairment in Psychiatry (SCIP), Confirmatory factor analysis, Structural equation modeling, Psychometrics

## Abstract

**Background:**

Psychiatric disorders, especially schizophrenia, are characterised by cognitive impairment. The rapid detection of cognitive dysfunction - also in the course of the disease - is of great importance. The Screen for Cognitive Impairment in Psychiatry (SCIP) was developed to provide screening of psychiatric patients in clinical practice and is available in several languages. Prior psychometric investigations into the dimensionality of the SCIP have produced two different models: a one-factor model assumes that the five subscales of the SCIP load together, whereas an alternative model suggests that the subscales load on two factors, namely verbal memory and processing speed. We carried out a confirmatory factor analysis of the German version of the SCIP (SCIP-G).

**Methods:**

323 patients with psychotic, bipolar affective, and depressive disorders were studied.

**Results:**

The one-factor approach did not yield an acceptable model fit (chi-squared test: χ^2^ = 109.5, df = 5, *p* < 0.001, χ^2^/df = 21.9). A two-factor solution, with the subtests Verbal Learning Test-Immediate Recall, Delayed Recall Test of the VLT, and Working Memory Test loading on the first factor, whereas the subtests Verbal Fluency Test and Psychomotor Speed Test loading on the second factor, obtained a good model fit (χ^2^ = 6.7, df = 3, *p* = 0.08, χ^2^/df = 2.2).

**Conclusions:**

These data show that a good model fit can be achieved with a two-factor solution for the SCIP. This study is the first to conduct a confirmatory factor analysis using the German SCIP version and to test its dimensional structure using a hypothesis-testing approach.

## 1 Introduction

Cognitive impairment is a key feature of patients with schizophrenia, involving deficits in attention, information processing, executive function, language and in memory, especially in verbal learning (Green and Harvey, 2014). Cognitive dysfunction can already manifest itself during the initial manifestation of schizophrenia (Furtner et al., 2021); it often persists in the course even after acute symptoms have subsided (Green et al., 2004) and can influence the functional outcome in schizophrenia as well as in affective disorders (Sachs et al., 2020). Despite such findings, screening for cognitive deficits frequently still is insufficient in routine clinical practice.

Various tests are available for the rapid assessment of cognitive dysfunction. In addition to the Brief Assessment of Cognition in Schizophrenia, which was created specifically for schizophrenia (BACS: Keefe et al., 2004, Sachs et al., 2011) and its further developments designed for testing non-schizophrenic patients as well (BAC, BAC-A, BAC-SF and BAC App: Keefe et al., 2014, Atkins et al., 2017, Lam et al., 2017), the Screen for Cognitive Impairment in Psychiatry (SCIP: Purdon, 2005) is available, which takes about 15-20 minutes. Apart from a pen, a test sheet, and a wristwatch, no other tools are needed, making this screening instrument suitable for routine examination in clinical psychiatry (Purdon, 2005). The screening provides initial indications for cognitive deficits in psychiatric disorders (including schizophrenia and affective disorders), which can quickly determine the need for further assessment (Purdon, 2005). The SCIP consists of five subtests: the Verbal Learning Test-Immediate Recall (VLT-I), the Delayed Recall Test of the VLT (D-VLT), the Working Memory Test (WMT), the Verbal Fluency Test (VFT), and the Psychomotor Speed Test (PST). For the SCIP, there are three parallel forms, which are regarded as equivalent and thus enable the recording of changes over time (Purdon, 2005; Pino et al., 2006, Pino et al., 2008; Guilera et al., 2009; Sachs and Erfurth, 2021; Sachs et al., 2021; Tourjman et al., 2016); in addition, unintentional learning effects from repeated testing are avoided.

The SCIP, originally devised in English, has been translated into many languages, including Chinese, Danish, French, German, Italian, Japanese, Persian (Farsi), Persian, Portuguese, Russian, and Spanish (Purdon, 2005; Pino et al., 2006; Hirabayashi et al., 2006; Jensen et al., 2015; Tourjman et al., 2016; Banazadeh et al., 2017; Belvederi Murri et al., 2020a; Belvederi Murri et al., 2020b; Sachs et al., 2021). To evaluate the underlying assumption of the SCIP that the test instrument can detect cognitive impairments, the dimensional structure has been analysed for the English, Spanish, French, and Italian versions, using principal component analyses or exploratory factor analyses (EFA) throughout. These analytic approaches have generally produced two different models.

The one-factor model assumes that the five SCIP subscales load together on the factor Cognitive Impairment. This model has been supported in investigations of the Spanish, Danish, and French SCIP versions (Cuesta et al., 2011; Gómez-Benito et al., 2014, Gómez-Benito et al., 2018; Pino et al., 2008; Guilera et al., 2009; Ott et al., 2016; Tourjman et al., 2016).

As for the two-factor model, two variants have emerged. The first one is based on the results of Belvederi Murri et al. (2020b), in which exploratory factor analysis suggested that the VLT-I, VLT-D, and WMT are related to the factor verbal memory, while the WMT, VFT, and PST load on another factor, namely processing speed. When evaluating this outcome, the problem arises that in confirmatory factor analysis (CFA) it is assumed that variables should load on a single factor (McDonald, 1985). However, with the WMT loading on both factors, this variant is characterised by a cross-loading pattern. Consequences of such cross-loadings include problems with discriminant validity, by inflating the correlations between factors and causing the loadings to be misrepresented (Marsh et al., 2020). Pino et al. (2006) obtained a two-factor model for the English SCIP version, with the first factor accounting for 38.92% (VLT-I, VLT-D, WMT) and the second factor for 23.45% (VFT, PST) of test variance. Similarly, the Spanish SCIP version showed two factors with the same allocation of subtests. Here, the first factor accounted for 39.20% and the second factor for 24.78% of variance. In similar vein, in the French study by Tourjman et al. (2016), the analysis revealed two factors for version 1 and 2 of the French SCIP, “with 4 (VLT-I, VLT-D, VMT, VWT) and 3 (VLT-I, VLT-D, VMT) subtests respectively loading heavily” on the first factor.

The aim of this study was to scrutinize the psychometric properties and to conduct a structural (i.e., dimensionality) analysis of the SCIP by performing a CFA of the German version of the SCIP (Sachs et al., 2021). Specifically, a series of four hypotheses was tested: (1) all five variables (subtests) load on one factor (cognitive impairment); (2), alternatively, the five variables (subtests) load on two factors; (3) there is a particularly strong correlation between VLT-I and VLT-D; and (4), there is a strong correlation between WMT and VFT.

## 2 Methods and materials

### 2.1 Study participants and assessments

For this study, 323 patients from the 1st Department of Psychiatry and Psychotherapeutic Medicine, Klinik Hietzing, Vienna, were included. Up to May 2018, this department was located at the Otto-Wagner-Spital, Vienna. The sample analysed here was composed of routinely collected clinical data of adult psychiatric inpatients residing in a specific catchment area and for whom an acute admission had previously been indicated.

The diagnostic classification was done by specialists in psychiatry and psychotherapeutic medicine according to the Criteria for Research of the 10th revision of the International Statistical Classification of Diseases and Related Health Problems, ICD-10 (World Health Organization, 2011), and the cognitive tests were conducted by clinical psychologists with the SCIP-G (German version: Sachs et al., 2021). The clinical psychologists received training, and a supervisor checked data entry, dating, missing values, and the accuracy of the data collected.

From 2011 to 2020, adult inpatients with psychotic disorders (ICD-10 F2 diagnosis of schizophrenia, schizotypal and delusional disorders), bipolar disorders (ICD-10 F30/F31 diagnosis) or depression (ICD-10 F32/F33 diagnosis) were included. All patients spoke German fluently. For the sake of sample homogeneity, patients over 65 years of age were excluded because of the increased probability of age-related mild cognitive impairment (Harada et al., 2013).

The study was conducted in accordance with the ethical principles of the Declaration of Helsinki and Good Clinical Practice. The study protocol was approved by the Ethics Committee of the City of Vienna. All patients gave written informed consent to take part in this study.

### 2.2 Data analysis

The data were analysed using IBM SPSS Statistics 23, R Studio version 1.2.5001, and the R package Lavaan (latent variable analysis and structural equation modeling; Rosseel, 2012). Pearson correlations were utilized to determine which subtests correlate with each other. The Kaiser-Maier-Olkin (KMO) criterion and the Bartlett test were used to check for sphericity.

### 2.3 Confirmatory factor analyses

The Kolmogorov-Smirnov and Shapiro-Wilk tests were used to test the SCIP-G data (i.e., subtest scores) for normal distribution (Bortz and Lienert, 2008). A one-factor model and a two-factor model were tested and compared, based on the results of previous studies and utilizing CFA.

Form 1 of the SCIP-G (Sachs et al., 2021) was tested for factorial validity, using the German-language sample. For analysis, the data were transformed using the percent of maximum possible (POMP) score method of Cohen et al. (1999) to align the different score ranges of the subtests of the SCIP to a common metric, thus creating equivalence and making individual test results and group differences in correct proportions comparable (Moeller, 2015). Since an open number of points can be achieved in the fourth subtest and thus the overall score potentially is unlimited, the maximum value from the VFT subtest was set at 30 points, such that the overall score maximum for the SCIP-G was set at 124 points.

The CFA was then performed, whereby the robust maximum likelihood method was chosen as the parameter estimator, since non-normally distributed data often lead to poorer model fit and deviations can thus be compensated for (Moosbrugger and Kelava, 2012; Backhaus et al., 2018).

For model evaluation, the fit measures chi-squared test (χ^2^ value), the Root Mean Square Error of Approximation (RMSEA), the Comparative Fit Index (CFI), the Normed Fit Index (NFI) and the Akaike Information Criterion (AIC) were calculated (Moosbrugger and Kelava, 2012). In addition, the Standardised Root Mean Square Residual (SRMR) was applied (Kline, 2005).

## 3 Results

### 3.1 Descriptive statistics

Age, gender, and the assignment of patients to diagnostic groups according to the ICD-10 Criteria for Research (World Health Organization, 2011) are set out in Table 1. Mean values and standard deviations of the subscale scores of the first form of the SCIP-G are shown in Table 2.

**Table 1:** Demographic variables.

	N	323
Age		
	Mean (± SD)	37.72 (13.01)
	Range	18–64
	Median	37
Gender (%)		
	Female	179 (55.4)
	Male	144 (44.6)
Diagnostic group (%)		
	F2 (schizophrenia, schizotypal and delusional disorders)	119 (36.8)
	F30/31 (bipolar disorder)	69 (21.4)
	F32/33 (depression)	126 (39.0)
	Missing	9 (2.8)

N represents sample size, SD = standard deviation, the diagnoses were made according to the ICD-10 Criteria for Research (World Health Organization, 2011).

**Table 2:** Means, standard deviations, and correlations with confidence intervals

Variable	M	SD	1	2	3	4	5
1. SCIP-G total	69.91	12.53					
2. VLT-I	22.68	4.13	0.81⁎⁎ [0.77, 0.84]				
3. WMT	18.72	3.68	0.74⁎⁎ [0.69, 0.79]	0.52⁎⁎ [0.43, 0.59]			
4. VFT	12.11	4.28	0.69⁎⁎ [0.63, 0.75]	0.36⁎⁎ [0.26, 0.45]	**0.38**⁎⁎ [0.28, 0.47]		
5. VLT-D	7.15	2.33	0.69⁎⁎ [0.63, 0.74]	**0.70**⁎⁎ [0.64, 0.75]	0.40⁎⁎ [0.31, 0.49]	0.23⁎⁎ [0.13, 0.33]	
6. PST	9.34	3.01	0.66⁎⁎ [0.59, 0.72]	0.37⁎⁎ [0.27, 0.46]	0.36⁎⁎ [0.26, 0.45]	0.41⁎⁎ [0.31, 0.49]	0.35⁎⁎ [0.25, 0.44]

M = Mean, SD = Standard Deviation. VLT-I = Verbal Learning Test-Immediate, WMT = Working Memory Test, VFT = Verbal Fluency Test, VLT-D = Verbal Learning Test-Delayed, PST = Psychomotor Speed Test. 95% confidence interval for correlations in brackets. Values in bold refer to the correlations between the VLT-I-VLT-D and VFT-WMT subtests addressed in Hypotheses 3 and 4 respectively (see main text).

Pearson correlations.

^⁎^*p* < 0.05..

⁎⁎ *p* < 0.01.

The mean age of the patients was 37.7 years (standard deviation SD: 13.0). 55.4% of the patients were women. The ICD-10 Criteria for Research for F2 diagnoses (schizophrenia, schizotypal and delusional disorders) were met by 36.8% of patients, for F30/F31 by 21.4%, and for F32/F33 by 39.0% in the sample.

### 3.2 Confirmatory factor analysis

By means of confirmatory factor analyses, two different models were subsequently tested, following the results of prior related research. The Kaiser-Maier-Olkin criterion for measuring the suitability of the data for factor analysis supported the applicability of the sample data, with a value of 0.73 and with a p value of <0.001 for Bartlett's test. Significant results obtained for the Kolmogorov-Smirnov and Shapiro-Wilk tests suggested that the data were not normally distributed. To carry out the best possible analysis under these circumstances, the robust maximum likelihood estimation method was used. Since the scale intercorrelations showed that there was no exceptionally strong association between the VFT and WMT subtests (r = 0.38, p < 0.001; Hypothesis 4), only the correlated error variance between VLT-I and VLT-D (r = 0.70, p < 0.001; Hypothesis 3) was added to the models, in order to save degrees of freedom in the analysis and thus to achieve better model identification (Comrey and Lee, 2013). One variable per model per factor was fixed at unity and served as a marker variable, thus creating a metric for the latent variable (Hoyle, 2014).

#### 3.2.1 Model 1

This one-factor model assumed all five subtests loading onto a single factor (Hypothesis 1). In addition, correlated error variance between VLT-I and VLT-D was assumed. The examination of the robust measures showed that a factor structure differing from Model 1 should be assumed. Table 3 shows the standardised factor loadings. As can be seen in Table 4, the χ^2^ value reached 109.5 at df = 5. The p value was <0.001, which was below the cut-off value of 0.05 (Kline, 2005). The χ2/df value of 21.9 clearly exceeded the recommended cut-off value of 3.00 (Moosbrugger and Kelava, 2012). The RMSEA at 0.254 and the SRMR at 0.207 both were above the threshold for a good model fit of 0.08. The measures CFI at 0.754 (uncorrected at 0.771) and NFI at 0.748 also did not reach the suggested cut-off values (CFI ≥ 0.90, NFI ≥ 0.95) for good or moderate model fit (Kline, 2005; Moosbrugger and Kelava, 2012).

**Table 3:** Factor loadings for the one-factor model and the two-factor model.

Variable	M1		M2	
		F1		F2
VLT-I	0.075	0.718		
WMT	0.588	0.722		
VFT	0.628			0.636
VLT-D	0.131	0.560		
PST	0.631			0.640
Correlated error term VLT-I-VLT-D	0.673		0.520	

*N* = 323. M1 = one-factor model, M2 = two-factor model. F1 = memory, F2 = processing speed (Pino et al., 2006). VLT-I = Verbal Learning Test-Immediate, WMT = Working Memory Test, VFT = Verbal Fluency Test, VLT-D = Verbal Learning Test-Delayed, PST = Psychomotor Speed Test. Calculations utilised the robust maximum likelihood estimation method; factor intercorrelation between M1 and M2 is *r* = 0.797 (*p* < 0.001); values shown are fully standardised factor loadings with *p* < 0.001.

**Table 4:** Goodness-of-fit statistics.

Fit measures	One-factor model (M1)	Two-factor model (M2)
χ^2^	109.517	6.730
χ^2^/df	21.9	2.24
p value	<0.001	0.081
RMSEA	0.254	0.062
CFI	0.754	0.991
NFI	0.748	0.985
SRMR	0.207	0.021
AIC	12,928.049	12,825.843

N = 323; p < 0.05; df(M1) = 5; df(M2) = 3.

For model evaluation, the model-fit measures chi-squared test (χ^2^ value), Root Mean Square Error of Approximation (RMSEA), Comparative Fit Index (CFI), Normed Fit Index (NFI), Standardised Root Mean Square Residual (SRMR), and Akaike Information Criterion (AIC) were used.

In addition, we conducted separate confirmatory factor analyses for psychotic and depressive samples. The results show no relevant improvement in the fit of the one-factor model for both samples (Table 5).

**Table 5:** Goodness-of-fit statistics of the separated groups for the one-factor-model.

Fit measures	Psychotic sample	Depressed sample
χ^2^	43.657	56.610
χ^2^/df	8.73	11.32
p value	<0.001	<0.001
RMSEA	0.258	0.227
CFI	0.770	0.763
NFI	0.751	0.781
SRMR	0.221	0.180
AIC	4690.325	7924.766

N(psychotic) = 116; p < 0.05; df = 5; N(depressed) = 200; *p* < 0.05; df = 5. For model evaluation, the model-fit measures chi-squared test (χ^2^ value), Root Mean Square Error of Approximation (RMSEA), Comparative Fit Index (CFI), Normed Fit Index (NFI), Standardised Root Mean Square Residual (SRMR), and Akaike Information Criterion (AIC) were used.

#### 3.2.2 Model 2

The two-factor model (Hypothesis 2) with error variance between VLT-I and VLT-D (Hypothesis 3) was tested based on observations from prior related research on the dimensionality of the SCIP. In Model 2, VLT-I, VLT-D, and WMT loaded on the first factor, whereas VFT and PST on a second factor (Pino et al., 2006). For this model (see Table 3), the loadings were 0.72, 0.56, and 0.72 (VLT-I, VLT-D, WMT) for the first factor and 0.64 and 0.64 (PST, VFT) for the second one. This shows that almost 52% of the variance of VLT-I, about 31% of VLT-D, and about 52% of WMT are attributable to the first factor, whilst almost 41% of the variance of PST and VFT can be attributed to the second factor. The correlation of the factors was significant with r = 0.797 (p < 0.001). The error term correlation between VLT-I and VLT-D was 0.52 (p < 0.001), which could be explained by the fact that both subtests consist of the same scale (Urban and Mayerl, 2014) and both are intended to cover verbal memory. The fit indices consistently indicated moderate to good model fit. In contrast to the one-factor model, the χ^2^ test with χ^2^ = 6.7 at df = 3 showed a significant improvement with a p value of 0.081 (cut-off: p > 0.05). Also, the χ^2^/df value was within the acceptable fit range in this case, at 2.2 (Moosbrugger and Kelava, 2012).

Again, we additionally conducted separate CFAs for psychotic and depressed samples (Table 6). Here the result shows a very good model fit for the psychotic sample and an acceptable model fit for the depressed sample (Table 7).

**Table 6:** Factor loadings for the two-factor model with separated groups psychotic vs. depressed.

Variable		Psychotic sample			Depressed sample	
		F1	F2		F1	F2
VLT-I		0.756			0.666	
WMT		0.705			0.691	
VFT			0.578			0.624
VLT-D		0.611			0.500	
PST			0.809			0.547
Correlated error term VLT-I-VLT-D	0.468			0.533		

N(psychotic) = 116; N(depressed) = 200. F1 = memory, F2 = processing speed (Pino et al., 2006). VLT-I = Verbal Learning Test-Immediate, WMT = Working Memory Test, VFT = Verbal Fluency Test, VLT-D = Verbal Learning Test-Delayed, PST = Psychomotor Speed Test. Calculations utilised the robust maximum likelihood estimation method; factor intercorrelation between F1 and F2 (psychotic sample) is *r* = 0.731 (p < 0.001); factor intercorrelation between F1 and F2 (depressed sample) is *r* = 0.842 (*p* < 0.001); values shown are fully standardised factor loadings with p < 0.001.

**Table 7:** Goodness-of-fit statistics of the separated groups for the two-factor-model.

Fit measures	Psychotic sample	Depressed sample
χ^2^	2.785	6.118
χ^2^/df	0.928	2.039
p value	0.426	0.106
RMSEA	0.000	0.072
CFI	1.000	0.986
NFI	0.985	0.976
SRMR	0.025	0.025
AIC	4650.596	7879.184

N(psychotic) = 116; p < 0.05; df = 3; N(depressed) = 200; p < 0.05; df = 3. For model evaluation, the model-fit measures chi-squared test (χ^2^ value), Root Mean Square Error of Approximation (RMSEA), Comparative Fit Index (CFI), Normed Fit Index (NFI), Standardised Root Mean Square Residual (SRMR), and Akaike Information Criterion (AIC) were used.

#### 3.2.3 Baseline model

To complete the analyses, we tested the baseline model and performed a hierarchical comparison between the baseline model, the one-factor-model and the two-factor-model using the chi-square difference test (Table 8).

**Table 8:** Chi-square difference test.

	df	AIC	BIC	Chi-square	Chi-square difference	Probability (>Chi-square)
Two-factor-model	3	12,826	12,871	6.8995		
One-factor-model	6	13,076	13,110	262.6651	183.52	<2.2e-16***
Baseline model	10	13,286	13,286	481.1519	228.78	<2.2e-16***

df: Degrees of freedom. BIC: Bayesian Information Criterion.

Signif. codes: 0 ‘***’ 0.001 ‘**’ 0.01 ‘*’ 0.05 ‘.’ 0.1 ‘ ’ 1

## 4 Discussion

The present data show that a good model fit can be achieved with Model 2 (VLT-I, VLT-D, and WMT loading on one factor, and VFT and PST loading on another one). Our study is the first to conduct a confirmatory factor analysis using the German version of the SCIP and to test the dimensional structure of the SCIP using a hypothesis-testing analytic approach.

The first model assumed a unidimensional factor for all subtests. This assumption did not achieve an acceptable model fit. A two-factor solution with the subtests VLT-I, VLT-D, and WMT loading on the first factor and the subtests VFT and PST loading on the second factor (Pino et al., 2006) obtained a good model fit. The different factor solutions described in the literature may be the result of different levels of relative variability across subtest scores (Belvederi Murri et al., 2020b). It is also worth noting that so far only exploratory analyses have been conducted, with the underlying criteria and assumptions (and, in turn, statistical conclusion validity) being different from confirmatory analyses (Marsh et al., 2020).

According to Pino et al. (2006), the two factors identified correspond to two broad domains of cognitive skills. The first factor would stand for verbal memory and includes subtests of verbal learning and working memory. The second factor stands for processing speed, including the subtests of speed of information processing and verbal language processing. Pino et al. (2006) refer to a study by Nuechterlein et al. (2005), in which cognitive domains and their associated areas were specified.

When testing the assumption that the VLT-I and VLT-D scales (hypothesis 3) and the VFT and WMT scales (hypothesis 4) each have stronger mutual correlations than to the other scales, only the first assumption could be confirmed. Pearson correlation showed the strongest association between any subtests was 0.70 (p < 0.001), which was observed between VLT-I and VLT-D. In addition, the error variance calculated in the CFA between the two variables yielded a value of 0.52, which can be explained as systematic measurement error due to scale similarity (Urban and Mayerl, 2014). The correlation between VFT and WMT, on the other hand, was in the lower range at r = 0.38 (p < 0.001), which did not support the assumption. Similar results have been found in the investigations by Pino et al. (2008) and Guilera et al. (2009). As mentioned above, statistically it may be a systematic measurement error due to the similarity of the scales between VLT-I and VLT-D, which makes the correlation between these two scales stronger than those between other scales. This could also explain the lower correlation between WMT and VFT, as these subtests do not consist of the same items. However, when interpreting these results, the weak correlation could indicate that the two subtests capture different abilities that may belong as two subdomains to certain main areas of cognition. Lezak et al. (2012) discuss the main domain of working memory and executive skills which encompasses both subtests. Nuechterlein et al. (2005) group processing speed and verbal language processing together and see working memory as a separate domain.

In Kim et al. (2018), on the other hand, working memory is subordinate to executive functions, whereas processing speed is a subdomain of attention, and verbal memory would be attributed to the umbrella term of learning and memory. Miyake et al. (2000) also classify working memory as an executive function, although according to Fröhlich (2010) it is also a function of short-term memory and can therefore be rooted in the memory domain. This could also explain the tested model, in which WMT is attributed to the factor that describes memory, and PST and VFT are found under the factor processing speed. These and further differing classifications make it difficult to differentiate the subtests into precise areas. In fact, all cognitive abilities are interconnected, overlapping, and influence each other (Kim et al., 2018; Trivedi, 2006; Nuechterlein et al., 2005). Analyses in two large datasets (5414 bipolar I patients and 3942 schizophrenia patients) suggest that cognition is “best explained as a single latent trait applicable to people with schizophrenia and bipolar disorder” (Harvey et al., 2016) thereby confirming earlier indications from studies in schizophrenia (Keefe et al., 2006; Harvey et al., 2013). Thus, it can be hypothesised that with respect to cognitive function, a one-factor solution is more likely the larger the sample.

In summary, the SCIP measures areas of cognition that are impaired in psychiatric disorders, such as schizophrenia; hence, it is well suited as a screening tool for a first impression of cognitive impairment. In our study, a two-factor solution, with VLT-I, VLT-D, and WMT loading on the first factor, and the VFT and PST loading on the second factor, yields a good model fit.

## 5 Limitations

The non-normally distributed data and the sample heterogeneity in terms of different diagnoses might have impacted on results. On the other hand, cognitive impairment is found across different psychiatric diagnoses (which is what the SCIP was designed for), and differences in cognitive impairment are more quantitative than qualitative.

A further limitation is that for the standardisation of the data using the POMP score method, the open test VFT was limited to 30 points and thus also the total result of the SCIP, since the maximally achievable score of the scales is required for the POMP method. In the context of factor analysis, the CFA restriction that variables load on only one factor at a time and cross-loadings are to be avoided must be considered (Marsh et al., 2020). This condition is often regarded as unduly strict for the purpose of representing the underlying model. This requirement frequently results in erroneous estimates, poor model fit, and inflated factor intercorrelations, which, in turn, has the effect of diminishing discriminant validity (Marsh et al., 2020).

## CRediT authorship contribution statement

**Gabriele Sachs:** Conceptualization, Methodology, Supervision, Writing – original draft, Writing – review & editing. **Gloria Bannick:** Methodology, Formal analysis, Investigation, Writing – original draft, Writing – review & editing. **Eva I.J. Maihofer:** Investigation, Data curation. **Martin Voracek:** Conceptualization, Methodology, Supervision, Writing – original draft. **Scot E. Purdon:** Conceptualization, Writing – original draft. **Andreas Erfurth:** Conceptualization, Resources, Project administration, Supervision, Writing – original draft, Writing – review & editing.

## Declaration of competing interest

The authors declare no conflict of interest.

## References

[bb0005] Atkins A.S., Tseng T., Vaughan A., Twamley E.W., Harvey P., Patterson T., Narasimhan M., Keefe R.S. (2017). Validation of the tablet-administered Brief Assessment of Cognition (BAC app). Schizophr. Res..

[bb0010] Backhaus K., Erichson B., Plinke W., Weiber R. (2018).

[bb0015] Banazadeh N., Khalili N., Mazhari S., Anaridokht F. (2017). Validity and reliability of a Persian version of the Screen for Cognitive Impairment in Psychiatry Scale in patients with bipolar type one disorder. Afzalipour J.Clin.Res..

[bb0020] Belvederi Murri M., Folesani F., Costa S., Biancosino B., Colla C., Zerbinati L., Caruso R., Nanni M.G., Purdon S.E., Grassi L. (2020). Screening for cognitive impairment in non-affective psychoses: a comparison between the SCIP and the MoCA. Schizophr. Res..

[bb0025] Belvederi Murri M., Folesani F., Costa S., Morelli A.C., Scillitani V., Guaiana G., Biancosino B., Caruso R., Nanni M.G., Zerbinati L., Purdon S.E., Grassi L. (2020). Italian validation of the screen for cognitive impairment in psychiatry. Community Ment. Health J..

[bb0030] Bortz J., Lienert G. (2008).

[bb0035] Cohen P., Cohen J., Aiken L.S., West S.G. (1999). The problem of units and the circumstance for POMP. Multivar. Behav. Res..

[bb0040] Comrey A.L., Lee H.B. (2013).

[bb0045] Cuesta M.J., Pino O., Guilera G., Rojo J.E., Gómez-Benito J., Purdon S.E., Franco M., Martínez-Arán A., Segarra N., Tabarés-Seisdedos R., Vieta E., Bernardo M., Crespo-Facorro B., Mesa F., Rejas J. (2011). Brief cognitive assessment instruments in schizophrenia and bipolar patients, and healthy control subjects: a comparison study between the Brief Cognitive Assessment Tool for Schizophrenia (B-CATS) and the Screen for Cognitive Impairment in Psychiatry (SCIP). Schizophr. Res..

[bb0050] Fröhlich W.D. (2010).

[bb0055] Furtner J., Schöpf V., Erfurth A., Sachs G. (2021). An fMRI study of cognitive remediation in drug-naïve subjects diagnosed with first episode schizophrenia. Wien. Klin. Wochenschr..

[bb0060] Gómez-Benito J., Guilera G., Pino O., Tabarés-Seisdedos R., Martínez-Arán A. (2014). Comparing neurocognitive impairment in schizophrenia and bipolar I disorder using the Screen for Cognitive Impairment in Psychiatry Scale. Int. J. Clin. Health Psychol..

[bb0065] Gómez-Benito J., Berrío Á.I., Guilera G., Rojo E., Purdon S., Pino O. (2018). The Screen for Cognitive Impairment in Psychiatry: proposal for a polytomous scoring system. Int. J. Methods Psychiatr. Res..

[bb0070] Green M.F., Harvey P.D. (2014). Cognition in schizophrenia: past, present, and future. Schizophr.Res.Cogn..

[bb0075] Green M.F., Kern R.S., Heaton R.K. (2004). Longitudinal studies of cognition and functional outcome in schizophrenia: implications for MATRICS. Schizophr. Res..

[bb0080] Guilera G., Pino O., Gómez-Benito J., Rojo J.E., Vieta E., Tabarés-Seisdedos R., Segarra N., Martínez-Arán A., Franco M., Cuesta M.J., Crespo-Facorro B., Bernardo M., Purdon S.E., Díez T., Rejas J., Spanish Working Group in Cognitive Function (2009). Clinical usefulness of the Screen for Cognitive Impairment in Psychiatry (SCIP-S) scale in patients with type I bipolar disorder. Health Qual. Life Outcomes.

[bb0085] Harada C.N., Natelson Love M.C., Triebel K.L. (2013). Normal cognitive aging. Clin. Geriatr. Med..

[bb0090] Harvey P.D., Raykov T., Twamley E.W., Vella L., Heaton R.K., Patterson T.L. (2013). Factor structure of neurocognition and functional capacity in schizophrenia: a multidimensional examination of temporal stability. J. Int. Neuropsychol. Soc..

[bb0095] Harvey P.D., Aslan M., Du M., Zhao H., Siever L.J., Pulver A., Gaziano J.M., Concato J. (2016). Factor structure of cognition and functional capacity in two studies of schizophrenia and bipolar disorder: implications for genomic studies. Neuropsychology.

[bb0100] Hirabayashi E., Purdon S.E., Masuya J., Matsumoto Y., Okada S., Yamashiro N., Iimori M. (2006). The Japanese version of the Screen for Cognitive Impairment in Psychiatry: a preliminary study. Int. Clin. Psychopharmacol..

[bb0105] Hoyle R.H. (2014).

[bb0110] Jensen J.H., Støttrup M.M., Nayberg E., Knorr U., Ullum H., Purdon S.E., Kessing L.V., Miskowiak K.W. (2015). Optimising screening for cognitive dysfunction in bipolar disorder: validation and evaluation of objective and subjective tools. J. Affect. Disord..

[bb0115] Keefe R.S., Goldberg T.E., Harvey P.D., Gold J.M., Poe M.P., Coughenour L. (2004). The Brief Assessment of Cognition in Schizophrenia: reliability, sensitivity, and comparison with a standard neurocognitive battery. Schizophr. Res..

[bb0120] Keefe R.S., Bilder R.M., Harvey P.D., Davis S.M., Palmer B.W., Gold J.M., Meltzer H.Y., Green M.F., Miller D.D., Canive J.M., Adler L.W., Manschreck T.C., Swartz M., Rosenheck R., Perkins D.O., Walker T.M., Stroup T.S., McEvoy J.P., Lieberman J.A. (2006). Baseline neurocognitive deficits in the CATIE schizophrenia trial. Neuropsychopharmacology.

[bb0125] Keefe R.S., Fox K.H., Davis V.G., Kennel C., Walker T.M., Burdick K.E., Harvey P.D. (2014). The Brief Assessment of Cognition in Affective Disorders (BAC-A): performance of patients with bipolar depression and healthy controls. J. Affect. Disord..

[bb0130] Kim E.J., Bahk Y.C., Oh H., Lee W.H., Lee J.S., Choi K.H. (2018). Current status of cognitive remediation for psychiatric disorders: a review. Front.Psychiatry.

[bb0135] Kline R.B. (2005).

[bb0140] Lam M., Wang M., Huang W., Eng G.K., Rapisarda A., Kraus M., Kang S., Keefe R.S.E., Lee J. (2017). Establishing the Brief Assessment of Cognition - short form. J. Psychiatr. Res..

[bb0145] Lezak M.D., Howieson D.B., Bigler E.D., Tranel D. (2012).

[bb0150] Marsh H.W., Guo J., Dicke T., Parker P.D., Craven R.G. (2020). Confirmatory factor analysis (CFA), exploratory structural equation modeling (ESEM), and set-ESEM: optimal balance between goodness of fit and parsimony. Multivar. Behav. Res..

[bb0155] McDonald R.P. (1985).

[bb0160] Miyake A., Friedman N.P., Emerson M.J., Witzki A.H., Howerty A., Wager T.D. (2000). The unity and diversity of executive functions and their contributions to complex “frontal lobe” tasks: a latent variable analysis. Cogn. Psychol..

[bb0165] Moeller J. (2015). A word on standardization in longitudinal studies: don't. Front. Psychol..

[bb0170] Moosbrugger H., Kelava A. (2012).

[bb0175] Nuechterlein K.H., Barch D., Gold J., Goldberg T., Green M., Heaton R. (2005). Identification of separable cognitive factors in schizophrenia. Schizophr. Res..

[bb0180] Ott C.V., Bjertrup A.J., Jensen J.H., Ullum H., Sjælland R., Purdon S.E., Vieta E., Kessing L., Miskowiak K.W. (2016). Screening for cognitive dysfunction in unipolar depression: validation and evaluation of objective and subjective tools. J. Affect. Disord..

[bb0185] Pino O., Guilera G., Gómez J., Rojo E., Vallejo J., Purdon S.E. (2006). Escala breve para evaluar el deterioro cognitivo en pacientes psiquiátricos [A brief scale to assess cognitive impairment in psychiatric patients]. Psicothema.

[bb0190] Pino O., Guilera G., Rojo J.E., Gómez-Benito J., Bernardo M., Crespo-Facorro B., Cuesta M., Franco M., Martinez-Aran A., Segarra N., Tabarés-Seisdedos R., Vieta E., Purdon S., Rejas J. (2008). Spanish version of the Screen for Cognitive Impairment in Psychiatry (SCIP-S): psychometric properties of a brief scale for cognitive evaluation in schizophrenia. Schizophr. Res..

[bb0195] Purdon S.E. (2005).

[bb0200] Rosseel Y. (2012). lavaan: an R package for structural equation modeling. J. Stat. Softw..

[bb0205] Sachs G., Erfurth A. (2021). Prediction of functional outcome in bipolar disorder: effects of cognitive remediation and cognitive psychoeducational group therapy. Eur.Psychiatry.

[bb0210] Sachs G., Winklbaur B., Jagsch R., Keefe R.S. (2011). Validation of the German version of the Brief Assessment of Cognition in schizophrenia (BACS): preliminary results. Eur.Psychiatry.

[bb0215] Sachs G., Berg A., Jagsch R., Lenz G., Erfurth A. (2020). Predictors of functional outcome in patients with bipolar disorder: effects of cognitive psychoeducational group therapy after 12 months. Front.Psychiatry.

[bb0220] Sachs G., Lasser I., Purdon S.E., Erfurth A. (2021). Screening for cognitive impairment in schizophrenia: psychometric properties of the German version of the Screen for Cognitive Impairment in Psychiatry (SCIP-G). Schizophr.Res.Cogn..

[bb0225] Tourjman S., Beauchamp M., Djouini A., Neugnot-Cerioli M., Gagner C., Baruch P., Beaulieu S., Chanut F., Daigneault A., Juster R., Potvin S., Purdon S.E., Renaud S. (2016). French validation of the Screen for Cognitive Impairment in Psychiatry. Open J.Psychiatry.

[bb0230] Trivedi J. (2006). Cognitive deficits in psychiatric disorders: current status. Indian J. Psychiatry.

[bb0235] Urban D., Mayerl J. (2014).

[bb0240] Dilling H., Mombour W., Schmidt M.H., Schulte-Markwort E., World Health Organization (2011). Internationale Klassifikation psychischer Störungen: ICD-10 Kapitel V (F). Diagnostische Kriterien für Forschung und Praxis.

